# *Helicobacter pylori*infection and its association with anemia among adult dyspeptic patients attending Butajira Hospital, Ethiopia

**DOI:** 10.1186/s12879-014-0656-3

**Published:** 2014-12-09

**Authors:** Dargaze Kibru, Baye Gelaw, Agersew Alemu, Zelalem Addis

**Affiliations:** Butajira Zonal Hospital, Southern Nations, Nationalities and Peoples Region, Butajira, Ethiopia; Department of Medical Microbiology, School of Biomedical and Laboratory Sciences, University of Gondar, Gondar, Ethiopia; Deapartment of Medical MIcrobiology, School of Biomedical and Laboratory Sciences, University of Gondar, Gondar, Ethiopia

**Keywords:** Anemia, Butajira, Dyspepeptic, H.pylori

## Abstract

**Background:**

*Helicobacter pylori* infection is associated with anemia. Understanding the magnitude of *H.pylori* infection and its association with anemia is important in the management of anemic patients. The aim of this study was to assess the association between *H.pylori* infection and anemia among dyspeptic patients.

**Methods:**

A cross-sectional study was conducted in Butajira Hospital, Southern Ethiopia among 401 systematically dyspeptic patients. A structured questionnaire was used to collected data about the patient characteristics. Blood samples were analyzed for red blood cell parameters. Stool samples were assessed for the presence of *H.pylori* antigens and the presence of intestinal helminthes. Data were summarized in frequencies (%) and mean (SD) as appropriate. Chi-square test, logistic regression and independent t-tests were used in the analysis as needed. In all cases P-value <0.05 was considered as statistically significant.

**Results:**

The overall prevalence of *H. pylori* infection was 52.4% and it was significantly associated with age, presence of intestinal parasites, smoking habit, alcohol drinking habit and body mass index. The prevalence of anemia among *H.pylori* infected patients (30.9%) was significantly (P < 0.001) higher than uninfected patients (22.5%). The mean (SD) values of HGB, MCV, MCH, MCHC, HCT and RBC count was significantly different between *H.pylori* infected and uninfected patients.

**Conclusion:**

This study showed high prevalence of H.pylori infection among dyspeptic patients and this was associated with age and some behavioral characteristics of the patients. H.pylori infected patients showed high rate of anemia prevalence as compared to their H.pylori unifected counter parts. From this study it can be recommended that intervention activities related to the behavioral characteristics and prevention of intestinal parasitic infections should be in place. The cross sectional nature of the study has a limitation to show cause and effect associations and hence association between *H.pylori* infections with anemia need to be investigated in cohort type studies.

## Background

*Helicobacter pylori* infection has been recognized as one of the most common chronic bacterial infections in humans and infecting more than half of the population of the world. The overall prevalence is high in developing countries [1]. *H. pylori* infection is a worldwide problem but the prevalence varies from country to country [1],[2]. *H. pylori* infection is acquired in early childhood and becomes a chronic infection if left untreated [3]. The majority of infected people remain asymptomatic, and only small portions develop illness, usually in adulthood [4]. *H. pylori* cause upper gastrointestinal disease such as gastritis, peptic ulcer disease and also increase the risk of gastric cancer [5],[6]. Male gender, increasing age, shorter height, tobacco use, lower socioeconomic status, obesity, and lower educational status of the parents in studies conducted among children are proposed risk factors for infection [7].

Several studies suggested an association between *H. pylori* infection with iron deficiency and anemia [8],[9]. *H. pylori* infection and extra gastric manifestations, pernicious anemia (10) and idiopathic thrombocytopenic purpura have been reported [10],[11]. Active *H. pylori* infection was independently associated with iron deficiency and iron-deficiency anemia [12],[13] and presence of *H. pylori* infection is associated with a poorer response to oral iron therapy [14]. It has been suggested that eradication of *H. pylori* may result in improvement of anemia even without iron supplementation [14],[15].

It is hypothesized that *H. pylori*-associated with anemia is caused by both compromised absorption of bio-available iron in the context of hypochlorhydria [16], and the competing iron demands of *H. pylori* and the host [17],[18]. Most dietary iron is in the non-hemic ferric form, and an acidic intra-gastric pH is needed to reduce it to the ferrous form for absorption. This reaction is promoted by gastric acidity and ascorbic acid, which is thus considered the most potent regulator of iron absorption [19]. *H. pylori* a major cause of chronic superficial gastritis leading to atrophy of gastric glands and leading to decreased gastric acid secretion [20]. *H. pylori* need iron to thrive and it possesses a 19-kDa iron-binding protein resembling ferritin, that may play a role in storage of excessive iron by the bacteria [21]. Moreover, since these bacteria have a high turnover rate, a large amount of iron may be lost in stools in the form of dead bacteria [22]. *H. pylori* has been found more frequently in dyspeptic patients [23]. The aim of the current study was to investigate the relation between *H. pylori* infection and anemia among dyspeptic patients attending medical care at Butajira Hospital.

## Methods

### Study area

The study was conducted at Butajira hospital which is found in Butajira town, Gurage Zone, Southern Nations Nationalities, and People’s Region (SNNPR) located 135 km from the capital-city, Addis Ababa. The town lies on the average at 2,100 m above the sea level. Butajira hospital is a zonal hospital with 110 beds that gives health service for peoples living in Butajira and the surrounding rural kebeles. The hospital report shows that it gives health service for an average of about 250 patients per day at the outpatient department. The hospital catchment area population is estimated around 1.3 million.

### Study design and period

A cross-sectional prospective study was conducted from April to Jun 2013.

### Sample size and sampling technique

The sample size was determined by using single population proportion formula taking 53% prevalence of *H. pylori* infection among dyspeptic patients [24] and a marginal error of 5%. Accordingly the sample size was determined 382 but we also considered a 5% non-response rate so that the final sample size was 401.

Study subjects were included using systematic random sampling technique. In Butajira hospital an average of 18 dyspeptic patients attend the outpatient clinic each day. The total expected dyspeptic patients during the study period were estimated 792. When the total population was divided by the sample size, the sample interval was found 2 and every two dyspeptic patient were selected until a total of 401 samples obtained.

All adult (age ≥18 years) patients presented with dyspepsia complaint were included in the study. Among those patients who were voluntary to participate in the study, those who had any surgery and blood donation, previous stomach or small bowel surgery, those who took treatment for *H. pylori* within the last three month, and pregnant women excluded. The patients were excluded, due to any of the exclusion criteria, from the study following critical review of their medical charts.

### Data collection and laboratory methods

#### General characteristics of the study participants

A structured questionnaire was used to collect data on demographic (sex, age, monthly income, marital status, educational status), behavioral (smoking, alcohol intake, dietary habit) and physical (body mass index) characteristics of the study participants. BMI ≤ 18.5 kg/m^2^ was classified as underweight; BMI = 18.6–24.9 kg/m^2^ as normal weight; BMI = 25-29.9 kg/m^2^ as overweight; and BMI ≥ 30 kg/m^2^ as obese [25].

### Sample collection and analysis

About 3 ml of venous blood was collected and examined for hematological parameters using Sysmex K-21 hematology analyzer. Approximately three gram of stool sample was collected in a clean screw cupped plastic container and checked for the presence of *H. pylori* antigen using *H. pylori* Rapid Test Strip (Creative Diagnostics.). Portion of the stool sample was used to assess the presence of intestinal helminthes using formol ether concentration technique as per a standard procedure [26].

### Data analysis and interpretation

Data were entered and analyzed using SPSS version 16.0. Continuous variables were summarized using means (±SD) and categorical variables were summarized in frequencies (percentages). Association between the prevalence of anemia and *H.pylori* infection was assessed by χ2 tests. Anemia was defined according to the WHO definition as a hemoglobin concentration of < 12 g/dL in women, < 13 g/dL in men [27]. The difference in the mean values of RBC parameters between *H.pylori* positive and negative individuals was explored using independent sample T-test. Logistic regression was used to determine the effect of independent variables on the prevalence of *H.pylori* infection. In all case a 95% confidence interval was used and P-values less than 0.05 were considered as statistically significant.

### Ethical consideration

The study was commenced after ethically approved by the ethical review committee of the School of Biomedical and Laboratory Science, University of Gondar. Permission to conduct the study was also obtained from the hospital administration. Written informed consent was obtained from each study participant and the results were kept confidential. Any result that was necessary for the patient was communicated with the physician for appropriate management.

## Results

### Sociodemographic, behavioral and physical characteristics of the study participants

A total of 401 adult dyspeptic patients (148 males and 253 females) with a mean (±SD) age of 37.3 (±13.7) years participated in this study. Majority of them were rural residents (70%), married (72.8%), and illiterate (56.9%). The mean (±SD) body mass index of the study population was 20.4 (±2.3) kg/m^2^. During the time of data collection about 4.5% and 10.7% of the study participants had a habit of cigarette smoking and drinking alcohol respectively. An assessment on the food habits of the study participants revealed that 8.7%, 63.8% and 33.4% had a habit of eating meat, vegetables and eggs 1-3 days/week respectively. Intestinal parasites were identified in 30.7% of the study participants (Table 1).

**Table 1:** **Sociodemographic, behavioral and physical characteristics of the study participants**

Characteristics	Frequency (%)
Sex	
Female	253(63%)
Male	148(37%)
Age	
18-28	130(32.4%)
29-38	93(23.2)
39-48	79(19.7)
49-58	66(16.5)
59-68	33(8.2)
Residence	
Urban	120(30)
Rural	281(70)
Family income/month ETB	
<776	271(67.6%)
≥776	130(32.4%)
Marital status	
Married	292(72.8)
Single	72(18)
Widowed/divorced	37(9.2)
Educational status	
Never attend school	228(56.9)
Primary school	92(22.9)
Secondary school and above	81(20.2)
BMI (kg/m^2^)	
Undernourished	75(18.7)
Normal	315(78.6)
Overweight	11(2.7)
No of people in household	
<5	298(74.3)
>6	103(25.7)
Cigarette smoking	
Yes	18(4.5)
No	383(95.5)
Alcohol drink	
Yes	43(10.7)
No	358(89.3)
Meat consumption per week	
Not at all	366(91.3)
>1-3	35(8.7)
Egg consumption per week	
Not at all	267(66.6
>1-3	134(33.4)
Vegetable consumption/week	
Not at all	20(5)
1-3	256(63.8)
>4	125(31.2)
Intestinal parasite	
Negative	278(69.3)
Positive	123(30.7)

### Prevalence of *H.pylori*infection and associated risk factors

The overall prevalence of *H. pylori* infection was 52.4% (n = 210); 66.7% (n = 140) in females and 33.3% (n = 70) in males. The relative frequency of the infection was higher in the age group 39-48 (50.6%) and among urban dwellers (54.4%). Among the different characteristics of the study participants’; age, presence of intestinal parasites, smoking habit, alcohol drinking habit and BMI showed statistically significant association with *H.pylori* infection (Table 2).

**Table 2:** **Bivariate and multivariate analysis of patient characteristics with** ***H. pylori*** **infection**

Risk factors	***H.pylori*** status		COR (95%CI)	P-value	AOR(95%CI)	P-value
	+ve	-ve				
Age category						
18-28	47	83	1		1	
29-38	44	49	1.58(0.92-2.72)	.095	1.37(0.77-2.45)	.277
39-48	40	39	1.81(1.03-3.20)	.040	1.74(0.95-3.17)	.070
49-58	49	17	5.09(2.64-9.82)	.000	5.18(2.58-10.42)	.000
59-68	30	3	17.66(5.11-60.2)	.000	17.63(4.95-62.7)	.000
Intestinal helminthes						
Negative	132	146	1		1	
Positive	78	45	1.92(1.24-2.96)	.003	1.9(1.17-3.07)	.009
Smoking						
Yes	16	2	7.89(1.77-34.36)	.007	8.36(1.68-41.64)	
No	194	189	1		1	
Alcohol drink						
Yes	18	25	0.62(0.33-1.18)	.15	0.37(0.17-0.82)	.014
No	192	166	1		1	
BMI category						.
Undernourished	53	22	2.00(0.55-7.27)	0.28	3.16(0.75-13.39)	.119
Normal	151	164	0.77(0.23-2.56)	0.67	1.10(0.28-4.29)	.889
Overweight	6	5	1		1	
Anemia						
Anemic	50	58	0.637(0.38-1.04)	0.072	1.58(0.96-2.61)	0.072
Non anemic	160	133	1		1	

### Prevalence of anemia and its association with *H.pylori*infection

Prevalence of anemia among dyspeptic patient was 26.9% (n = 108); 64.8% in females and 35.2% in males. The mean (±SD) hemoglobin concentration was 13.2(±1.4) g/dl and 14.1(±1.5) g/dl in females and males respectively. The prevalence of anemia among *H.pylori* infected patients was 30.9% and 22.5% among uninfected patients. The difference in the prevalence of anemia between *H.pylori* infected and uninfected patients was statistically significant (χ2 = 26.8; P < 0.001) (Table 3). The mean (SD) of other parameters related to red blood cell were also compared between *H.pylori* infected and uninfected patients. Accordingly statistically significant differences were observed in HGB, MCV, MCH, MCHC, HCT and number of RBC (Table 4).

**Table 3:** **Association between H.pylori sero-status and anemia prevalence among dyspeptic patients**

	H.pylori sero-positive	H.pylori sero-negative	***χ*** 2 ( ***P-value*** )
Anemic	65 (30.95)	43 (22.5)	3.62 (0.05)
Non anemic	145 (69.01)	148 (77.5)

**Table 4:** **Association between RBC indices with** ***H.pylori*** **infection**

Parameter	Mean (SD)		P-value (95% CI)
	H.pylori positive	H.pyori negative	
HGB	13.3(1.3)	13.8 (1.6)	0.001(0.19, 0.77)
MCV(fl)	87.5(7.2)	88.9(7.3)	0.058 (-0.048, 2.79)
MCH	27.85(2.54)	28.65(2.78)	0.003 (0.28, 1.33)
MCHC (ρg)	31.35(2.03)	32(2.33)	0.002 (0.26, 1.12)
RBCX10^6^/μl	4.63(0.59)	4.8(0.72)	0.01 (0.04, 0.29)
HCT (%)	41.9(5.3)	43.3(5.9)	0.009 (0.38, 2.58)

HGB = hemoglobin; MCV = mean corpuscular volume; MCH = mean corpuscular hemoglobin; MCHC = mean corpuscular hemoglobin concentration; RBC = red blood cell; HCT = hematocrit.

## Discussion

In this study, the prevalence of *H. pylori* infection among patients with dyspeptic symptoms was 52.37%. This prevalence is relatively lower than other reports conducted in different parts of African and Asia continent, which reported a prevalence ranging from 67% up to 86.8% [28]-[31]. The prevalence of *H pylori* varies greatly among countries and among population groups within the same country [2]. However, our finding is relatively similar with previous reports made in Ethiopia 53% [24] and Kuwait 49.7% [32]. Lack of clear cut definition of dyspepsia, *H. pylori* diagnostic method, sample size, social and economic factors could be some of the possible reasons for these variations.

The results of the current study also showed slight difference in prevalence of *H. pylori* infection between females and males (55.3% vs 47.3% respectively), but the difference was not statistically significant (P = 0.12). This finding goes in contrary to previous reports that indicated females were at significant risk to have for *H. pylori* infection (24, 32,33). However, our findings agree with other studies that showed the rate of *H. pylori* infection is independent of gender [31],[24],[30].

We found correlation between age and *H. pylori* infection being the prevalence was higher in older age groups (P < 0.001). This finding is in accordance with the results of former studies made in Kuwait [32] South Africa [33] and Ethiopia [30]. Moreover, study conducted in Addis Ababa, Ethiopia, showed a peak prevalence of *H. pylori* infection among older patients, within the age group between 54-61 years [24]. The most probable reason is that infection by *H. pylori* can be acquired in earlier age and persist throughout the life time of the patient and may cause disease at older age. However, there are also reports that showed higher prevalence of *H. pylori* infection during the younger age. For example, study conducted in Iran showed patient at younger age were more affected [31] and in Nigeria the peak prevalence of *H. pylori* infection was found among patients within the age group between 20-39 years old [29].

The current study result also showed a significant negative association betteween alcohol consumption and *H. pylori* infection (AOR 0.37; 95%; CI 0.17-0.82, P = 0.014). This result contradicts with previous report from Gondar, Ethiopia [30] and South Africa [33] that showed a positive association between *H. pylori* infection and alcohol consumption. In those studies it was reported that alcohol consumption could be a risk factor for *H. pylori* infection. Nevertheless, there are also reports that documented a non statistical risk reduction of *H. pylori* infection upon alcohol consumption [34]. Besides, the type and amount of alcohol had also an effect on the association. However, basic microbiology tells us that alcohol is known to have direct antimicrobial effects. Therefore, the lower prevalence of *H. pylori* infection among patients that consumed alcohol compared with the non-alcoholics attracted us to support the hypothesis that alcohol intake may have preventive effect for *H. pylori* infection.

In this study cigarette smoking was significantly associated with *H. pylori* infection (P = 0.01). Unlike other studies that reported no significant association with current smoking or any other measure of using tobacco [34]. Others proposed that smoking appears to affect treatment success [35]. These contradictory results may be due to uncontrolled confounding factors such as social class or differential antibiotic use.

Intestinal parasitic infection in this study was significantly associated with *H. pylori* infection (p = 0.009). This is different from a finding from Australia [36]. Intestinal parasitic infections and elevated IgE levels were associated with a reduced *H. pylori* prevalence in adults, living in Mexico, suggesting that intestinal parasites could affect persistence of *H. pylori* [37]. The presence of association in our study may be due to poor hygienic status that favors high rate of parasitic infection and similar route of transmission shared by *H.pylori*. But the real mechanism of interaction needs to be investigated with cohort studies.

There are quite a number of studies in the literature demonstrated the relationship between *H. pylori* infection and anemia. In the current study, the prevalence of anemia among *H. pylori* positive patients (n = 65, 30.95%) was significantly higher (P = 0.05) than *H .pylori* negative patients (n = 43; 22.5%). But other studies from Latin American countries showed no association [38] while a study from Haiti showed an inverse association [39]. The association observed in our study was also reflected on other RBC parameters as determined using *t*-test. We found that *H. pylori* stool antigen positive patients have significantly lower hemoglobin and hematocrit levels than *H. pylori* negative patients (13.3 g/dl versus 13.8 g/dl, P = 0.001) and (41.9% versus 43.3%, P = 0.009) respectively. Similar observation was reported from Turkish among teenager [40]. However, findings are not in agreement with the reports made by Fraser *et al* [41] and Kermati *et al* [42] where no association between *H. pylori* infection and Hgb/HCT levels was reported. Moreover, there was statistically significant difference in MCHC (P = 0.002) and MCH (P = 0.003) values between H*. pylori* stool antigen positive and negative dyspeptic patients, indicating the impact of *H. pylori* infection on hematological parameters.

### Limitations of the study

This study was conducted on dyspeptic patients who have had many underline disease conditions as a confounding factors that could not fully controlled so that it might have impact on the outcome of the statistical correlations between *H. pylori* infection, anemia and other variables of interest. The cross sectional nature of the study was also another limitation to show cause and effect relationship between the variables.

## Conclusion

This study indicated that the prevalence of *H. pylori* infection was high among dyspeptic patients in the study area. The rate of H*. pylori* infection was also increasing in advancing age showing that age is one of the risk factors in acquiring the infection. Moreover, cigarette smoking, and intestinal helmintic infection were identified as risk factors for *H. pylori* infection too. Alcohol consumption habit in our study was negatively associated with *H. pylori* infection. Mean hematological parameters and RBC were significantly reduced among *H. pylori* positive patients compared. This study indicated the need for further large scale study to determine the possible risk factors for such high rate of infection. Moreover cohort type studies are recommended to formulate a cause and effect relationship between the risk factors and *H.pylori* sero-positivity.
